# Supplementary material for: Metabolite‐sensitive cross‐bridge models of human atria reveal the impact of diabetes on muscle mechanoenergetics
Source: J Physiol. 2025 Jul 27;604(13):5316–41. doi: 10.1113/JP288463 (PMC13327773; doi:10.1113/JP288463)
Supplement: Supplementary file 1 — Peer Review History [file TJP-604-5316-s001.pdf]

The Journal of Physiology

Metabolite-sensitive cross-bridge models of human atria reveal the impact of diabetes on muscle mechanoenergetics

Julia H Musgrave, June-Chiew Han, Marie-Louise Ward, Andrew Taberner, and Kenneth Tran
DOI: 10.1113/JP288463

Corresponding author(s): Julia Musgrave (julia.musgrave@auckland.ac.nz)

The following individual(s) involved in review of this submission have agreed to reveal their identity: Kenneth S. Campbell (Referee #1)

Review Timeline:

Submission Date:	01-Mar-2025
Editorial Decision:	31-Mar-2025
Revision Received:	30-May-2025
Accepted:	13-Jun-2025

Senior Editor: Bjorn Knollmann

Reviewing Editor: Axel Loewe

Transaction Report:

(Note: With the exception of the correction of typographical or spelling errors that could be a source of ambiguity, letters and reports are not edited. Depending on transfer agreements, referee reports obtained elsewhere may or may not be included in this compilation. Referee reports are anonymous unless the Referee chooses to sign their reports.)

Dear Dr Musgrave,

Re: JP-RP-2025-288463 "Metabolite-sensitive cross-bridge models of human atria reveal the impact of diabetes on muscle mechanoenergetics" by Julia H Musgrave, June-Chiew Han, Marie-Louise Ward, Andrew Taberner, and Kenneth Tran

Thank you for submitting your manuscript to The Journal of Physiology. It has been assessed by a Reviewing Editor and by 2 expert referees and we are pleased to tell you that it is potentially acceptable for publication following satisfactory major revision.

Please advise your co-authors of this decision as soon as possible.

The referee reports are copied at the end of this email.

Please address all the points raised and incorporate all requested revisions or explain in your Response to Referees why a change has not been made. We hope you will find the comments helpful and that you will be able to return your revised manuscript within 2 months. If you require longer than this, please contact journal staff: jp@physoc.org. Please note that this letter does not constitute a guarantee for acceptance of your revised manuscript.

Your revised manuscript should be submitted online using the link in your Author Tasks: Link Not Available. This link is accessible via your account as Corresponding Author; it is not available to your co-authors. If this presents a problem, please contact journal staff (jp@physoc.org). Image files from the previous version are retained on the system. Please ensure you replace or remove any files that are being revised.

If you do not wish to submit a revised version of your manuscript, you must inform our journal staff (jp@physoc.org) or reply to this email to request withdrawal. Please note that a manuscript must be formally withdrawn from the peer review process at one journal before it may be submitted to another journal.

TRANSPARENT PEER REVIEW POLICY: To improve the transparency of its peer review process, The Journal of Physiology publishes online as supporting information the peer review history of all articles accepted for publication. Readers will have access to decision letters, including Editors' comments and referee reports, for each version of the manuscript, as well as any author responses to peer review comments. Referees can decide whether or not they wish to be named on the peer review history document.

ABSTRACT FIGURES: Authors are expected to use The Journal's premium BioRender account to create/redraw their Abstract Figures. Information on how to access this account is here: https://physoc.onlinelibrary.wiley.com/journal/14697793/biorender-access.

This will enable Authors to create and download high-resolution figures. If authors have used the free BioRender service, they can use the instructions provided in the link above to download a high-resolution version suitable for publication.

The link provided should only be used for the purposes of this submission. Authors will be charged for figures created on this account if they are not related to this manuscript submission.

LANGUAGE EDITING AND SUPPORT FOR PUBLICATION: If you would like help with English language editing, or other article preparation support, Wiley Editing Services offers expert help, including English Language Editing, as well as translation, manuscript formatting, and figure formatting at www.wileyauthors.com/eeo/preparation. You can also find resources for Preparing Your Article for general guidance about writing and preparing your manuscript at www.wileyauthors.com/eeo/prepresources.

RIGOUR AND REPRODUCIBILITY: We recommend authors consult The Journal's Rigour and Reproducibility requirements to ensure that their manuscript is suitable for publication: https://physoc.onlinelibrary.wiley.com/pb-assets/hub-assets/physoc/documents/TJP-Rigour-and-Reproducibility-Requirements.pdf

REVISION CHECKLIST:

Check that your Methods section conforms to journal policy: https://jp.msubmit.net/cgi-bin/main.plex?form_type=display_requirements#methods.

Check that data presented conforms to the statistics policy: https://jp.msubmit.net/cgi-bin/main.plex?form_type=display_requirements#statistics.

Upload a full Response to Referees file. To create your 'Response to Referees': copy all the reports, including any comments from the Senior and Reviewing Editors, into a Microsoft Word, or similar, file and respond to each point, using font or background colour to distinguish comments and responses and upload as the required file type.

Please upload two versions of your manuscript text: one with all relevant changes highlighted and one clean version with no

changes tracked. The manuscript file should include all tables and figure legends, but each figure/graph should be uploaded as separate, high-resolution files.

You may also upload:

- 'Potential Cover Art' for consideration as the issue's cover image
- Appropriate Supporting Information (Video, audio or data set: see https://jp.msubmit.net/cgi-bin/main.plex?form_type=display_requirements#supp).

We look forward to receiving your revised submission.

If you have any queries, please reply to this email and we will be pleased to advise.

Yours sincerely,

Bjorn Knollmann
Senior Editor
The Journal of Physiology

REQUIRED ITEMS

- Author photo and profile. First or joint first authors are asked to provide a short biography (no more than 100 words for one author or 150 words in total for joint first authors) and a portrait photograph. These should be uploaded and clearly labelled together in a Word document with the revised version of the manuscript. See Information for Authors for further details.

- The reference list must be in alphabetical order, rather than numbered, to comply with our Journal format.

- Please upload separate high-quality figure files via the submission form.

- Please ensure that any tables are editable and in Word format, and wherever possible, embedded in the article file itself.

- Please ensure that the Article File you upload is a Word file.

- Please include an Abstract Figure file, as well as the Figure Legend text within the main article file. The Abstract Figure is a piece of artwork designed to give readers an immediate understanding of the research and should summarise the main conclusions. If possible, the image should be easily 'readable' from left to right or top to bottom. It should show the physiological relevance of the manuscript so readers can assess the importance and content of its findings. Abstract Figures should not merely recapitulate other figures in the manuscript. Please try to keep the diagram as simple as possible and without superfluous information that may distract from the main conclusion(s). Abstract Figures must be provided by authors no later than the revised manuscript stage and should be uploaded as a separate file during online submission labelled as File Type 'Abstract Figure'. Please also ensure that you include the figure legend in the main article file. All Abstract Figures should be created using BioRender. Authors should use The Journal's premium BioRender account to export high-resolution images. Details on how to use and access the premium account are included as part of this email.

EDITOR COMMENTS

Reviewing Editor:

Ethics Concerns:
While data were reused from a previous study and not acquired in the scope of the work presented here, details on informed consent, IRB approval etc. should be stated.

Comments to the Author (Required):
Your manuscript was reviewed by two expert referees who acknowledge that this is an interesting study addressing a relevant question. In their reports, both referees point out a number of points that need to be addressed for further consideration of this manuscript including specification of the key points.

Please also see 'Required Items' above.

Senior Editor:

I concur with the reviewing editor. The manuscript has merit, but will require substantial revisions before it can be considered for publication.

REFEREE COMMENTS

Referee #1:

This is a well-written comprehensive manuscript that describes mathematical models of atrial myocardial tissue from patients with and without diabetes. The models were consistent with experimental results showing slower cross-bridge cycling and lower force production in tissue from patients with diabetes. These may reflect an increased proportion of beta myosin heavy chain in atrial tissue from patients with diabetes.

There is a lot to like about this paper. It presents state-of-the-art simulations that integrate well with a prior impressive experimental study.

This reviewer has minimal comments.

Fig 3 - The Ca50 of 0.33 µM for the non-diabetic group seems problematic here. Does this not imply that the myocardium is markedly affected even during diastole?

Fig 6 - relaxation in all conditions seems slow relative to that typically shown in a Wiggers diagram. Can the authors comment?

Finally, it would have been nice to see experimental confirmation of the change in myosin heavy composition. This reviewer realizes that this might not be within the researcher's skillset but it could easily be tested by new collaborators.

Referee #2:

This manuscript presents novel metabolite-sensitive mathematical models for non-diabetic and diabetic human atrial cross-bridge function, integrating these into whole-muscle models to examine mechanoenergetic effects of diabetes. Models reveal mechanisms behind reduced force and slower cross-bridge cycling in diabetic muscles, with downstream effects on twitch duration, power, and efficiency. Provides mechanistic insights and suggests therapeutic targets. Methods and findings are sound overall.

Comments and suggestions:

- The key points are vague and non-specific, limiting their usefulness. The first two points are general statements not specific to your study. Point three mentions "confirming mechanisms" without stating what these mechanisms are. Point four refers to "these differences" without clarity on which differences. Point five claims to offer "clear mechanisms" without naming them. This vagueness obscures the mechanistic insights revealed by the modeling work, making the manuscript appear more methodologically focused than it is. Reformulate the key points to explicitly state the specific mechanisms and findings you've discovered.

- For example, the authors could think of mentioning the identification of slower detachment rates (particularly k_3 and k_{-1}) and reduced cross-bridge stiffness (lower K) underlying diabetic cardiac dysfunction at the cross-bridge level. Or consider to emphasize the hypothesis generating value of the work, e.g. the signs of differential metabolite sensitivities in diabetic tissue (reduced P_1 sensitivity but increased ATP sensitivity), with implications for energetic dysfunction.

- Have you performed parameter identifiability analysis? Parameter correlations or profile likelihood approaches would strengthen confidence in your 11-parameter model. Without such analysis, it remains unclear whether alternative parameter sets might explain the experimental observations equally well, potentially leading to different mechanistic interpretations of diabetic cardiac dysfunction.

- The authors do not explicitly discuss boundary conditions or constraints to ensure physiological behavior of the model, particularly for the strain-dependent exponential terms in Equations 9 and 12.

- The model does not include the more recently characterized myosin structural states like SRX and DRX that represent different energy-consuming conformations of detached myosin heads. This is a simplification that may affect the model's ability to fully capture energetic aspects of myosin function, particularly since the SRX state is associated with reduced ATP consumption and has been implicated in several cardiomyopathies. Given the paper's focus on mechanoenergetics, the potential relevance of SRX/DRX dynamics to diabetes-related cardiac dysfunction deserves a discussion.

- The authors reject rapid equilibrium binding for P_1 citing "poorer fits" without quantifying this difference or providing mechanistic rationale for why direct incorporation at k_{-1} is more appropriate than at k_{-2}.

END OF COMMENTS

EDITOR COMMENTS

Reviewing Editor:

Ethics Concerns:
While data were reused from a previous study and not acquired in the scope of the work presented here, details on informed consent, IRB approval etc. should be stated.

We have included the ethics approval and patient consenting details at the end of the relevant Methods section (lines 63-66)

"The data in this study were obtained from patients undergoing routine cardiac surgery following provision of their informed consent for a small piece of right atrial tissue to be obtained for research purposes. The consenting process conformed to the principles outlined in the Declaration of Helsinki and was approved by the Human and Disability Ethics Committee of New Zealand (HDEC PR6432)"

Comments to the Author (Required):

Your manuscript was reviewed by two expert referees who acknowledge that this is an interesting study addressing a relevant question. In their reports, both referees point out a number of points that need to be addressed for further consideration of this manuscript including specification of the key points.

Please also see 'Required Items' above.

Senior Editor:

I concur with the reviewing editor. The manuscript has merit, but will require substantial revisions before it can be considered for publication.

REFEREE COMMENTS

Referee #1:

This is a well-written comprehensive manuscript that describes mathematical models of atrial myocardial tissue from patients with and without diabetes. The models were consistent with experimental results showing slower cross-bridge cycling and lower force production in tissue from patients with diabetes. These may reflect an increased proportion of beta myosin heavy chain in atrial tissue from patients with diabetes.

There is a lot to like about this paper. It presents state-of-the-art simulations that integrate well with a prior impressive experimental study.

This reviewer has minimal comments.

We thank the referee for their kind comments and the time taken to review our manuscript.

Fig 3 - The Ca50 of 0.33 μM for the non-diabetic group seems problematic here. Does this not imply that the myocardium is markedly affected even during diastole?

We acknowledge that the half-activation point of Ca^{2+} seems quite low, given the level of the diastolic [Ca^{2+}] shown in this figure. Both values came from the experimental study of Jones *et al.* (2023). In that study, the dynamic twitch data did not show elevated diastolic force. We have repeated the isometric simulations and can confirm that cross-bridge force accounts for less than 15 % of the total diastolic force (~ 0.78 kPa) in the non-diabetic muscle model. This corresponds to ~ 3.5 % of the maximal activation of the cross-bridge model. The non-diabetic myocardium is therefore not markedly affected during diastole.

Fig 6 - relaxation in all conditions seems slow relative to that typically shown in a Wiggers diagram. Can the authors comment?

While these work-loop simulations mimic in-vivo behaviour more closely than isometric contractions, we acknowledge that this not a direct analogue of the whole-heart work-loop. These simulations instead reflect the in-vitro experiments often performed in intact tissue preparations. While there are no equivalent experimental data available from human tissues, the non-diabetic work-loops are qualitatively similar to rat ventricular work-loops measured routinely in our lab (Han *et al.*, 2014). We note that the axes and normalisations of our plots emphasise the duration of the twitches (e.g. the myocyte twitch in Fig 5B appears to relax much quicker) but also recognise some factors that could contribute to a delayed relaxation.

In the non-diabetic model, the relaxation of stress follows the Ca^{2+} transient closely, so this can be seen as the main driver for relaxation rate. High-affinity Ca^{2+} indicators (such as fura-2, which was used to measure the Ca^{2+} transients that drive our simulations) may result in a prolongation of the measured transient duration (Kong & Fast, 2014). If our Ca^{2+} transients did have an increased duration, this would lead to simulations of slower twitch relaxation compared to what would be seen in intact tissues.

Regarding the work-loop simulations specifically, Chung *et al.* (2017) have shown in similar experiments that including a short re-stretch phase immediately after peak contraction will increase the relaxation rate. They suggest that this is what is experienced by myocytes within the heart, so this is a potential reason these types of experiments or simulations may

exhibit slower relaxation than a Wiggers diagram.

Finally, it would have been nice to see experimental confirmation of the change in myosin heavy composition. This reviewer realizes that this might not be within the researcher's skillset but it could easily be tested by new collaborators.

In our cited experimental paper, the change in myosin composition was confirmed by performing a SWATH-MS/MS analysis of diabetic and non-diabetic human atrial samples. The proportion of MYH6 (alpha myosin isoform) was significantly higher in the non-diabetic group. That this was measured in the referenced paper was not explicitly stated in the relevant discussion of the present paper, so we have adjusted it to make this clearer (see line 552).

“In Musgrave et al. (2025), a decrease in the proportion of a-myosin isoform was measured and identified as a likely mechanism underlying the observed slower cross-bridge cycling rates.”

Referee #2:

This manuscript presents novel metabolite-sensitive mathematical models for non-diabetic and diabetic human atrial cross-bridge function, integrating these into whole-muscle models to examine mechanoenergetic effects of diabetes. Models reveal mechanisms behind reduced force and slower cross-bridge cycling in diabetic muscles, with downstream effects on twitch duration, power, and efficiency. Provides mechanistic insights and suggests therapeutic targets. Methods and findings are sound overall.

We thank the referee for their kind comments and the time taken to review our manuscript.

Comments and suggestions:

- The key points are vague and non-specific, limiting their usefulness. The first two points are general statements not specific to your study. Point three mentions "confirming mechanisms" without stating what these mechanisms are. Point four refers to "these differences" without clarity on which differences. Point five claims to offer "clear mechanisms" without naming them. This vagueness obscures the mechanistic insights revealed by the modeling work, making the manuscript appear more methodologically focused than it is. Reformulate the key points to explicitly state the specific mechanisms and findings you've discovered.
- For example, the authors could think of mentioning the identification of slower detachment rates (particularly k_3 and k_{-1}) and reduced cross-bridge stiffness (lower K) underlying diabetic cardiac dysfunction at the cross-bridge level. Or consider to emphasize the hypothesis generating value of the work, e.g. the signs of differential metabolite sensitivities in diabetic tissue (reduced P_1 sensitivity but increased ATP sensitivity), with implications for energetic dysfunction.

Thank you for the feedback on our key points, including these helpful suggestions. Our key points have been reformulated to be more specific about the mechanisms we are referring to.

"-Cross-bridge function in the diabetic heart is important to understand, as it contributes to all active mechanical function in the heart and is affected by changes in metabolite availability.

-We developed mathematical models of non-diabetic and diabetic cross-bridges using data from human atrial tissues. Reduced cross-bridge attachment and detachment rates, and lower cross-bridge stiffness were identified as the key mechanisms underlying slower cross-bridge cycling and lower force production, respectively, in diabetic tissues.

-In simulations of isometric and work-loop contractions, these differences led to longer force twitches with lower amplitude, less work done, slower shortening and lower power generation in the diabetic muscles.

-The diabetic model predicted reduced sensitivity to inorganic phosphate but increased sensitivity to ATP, with implications in diabetic metabolic dysfunction.

-This modelling study presents novel human atrial cross-bridge models and identifies clear mechanisms behind mechanical and energetic dysfunction in diabetic heart muscle with potential pathways for treatment."

- Have you performed parameter identifiability analysis? Parameter correlations or profile

likelihood approaches would strengthen confidence in your 11-parameter model. Without such analysis, it remains unclear whether alternative parameter sets might explain the experimental observations equally well, potentially leading to different mechanistic interpretations of diabetic cardiac dysfunction.

We thank the referee for their suggestion of performing a profile likelihood analysis and have conducted one. Given its relevance to potential future users of this model, we have included this in the appendix following the full model equations:

"To assess how well-identified the parameters in the cross-bridge model were, we completed a profile likelihood analysis. Each of the 11 parameters in the non-diabetic model was changed in increments between half and double the final optimal value we identified (Table 3). This parameter was held fixed while all others were allowed to vary to find a new optimal fit using our parameter fitting protocol described in the methods. The objective function value for each optimal fit was plotted against changes in the value of each parameter (Figure A1). Most of the variables were found to have good identifiability. That is, as we vary the value of the parameter around its optimal value, the objective function value increased. Note that in this analysis, doubling of parameter values meant that two of the parameters (ϕ_l and K) were assigned values outside the range that was allowed in the original optimisation. As discussed in Section 2.2.2, the upper bound of K was constrained so that proportions of attached cross-bridges were similar in the two models. The upper bound for ϕ_l is consistent with previous work (Musgrave et al., 2024) and maintains sensible fractions of available cross-bridges at resting sarcomere lengths."

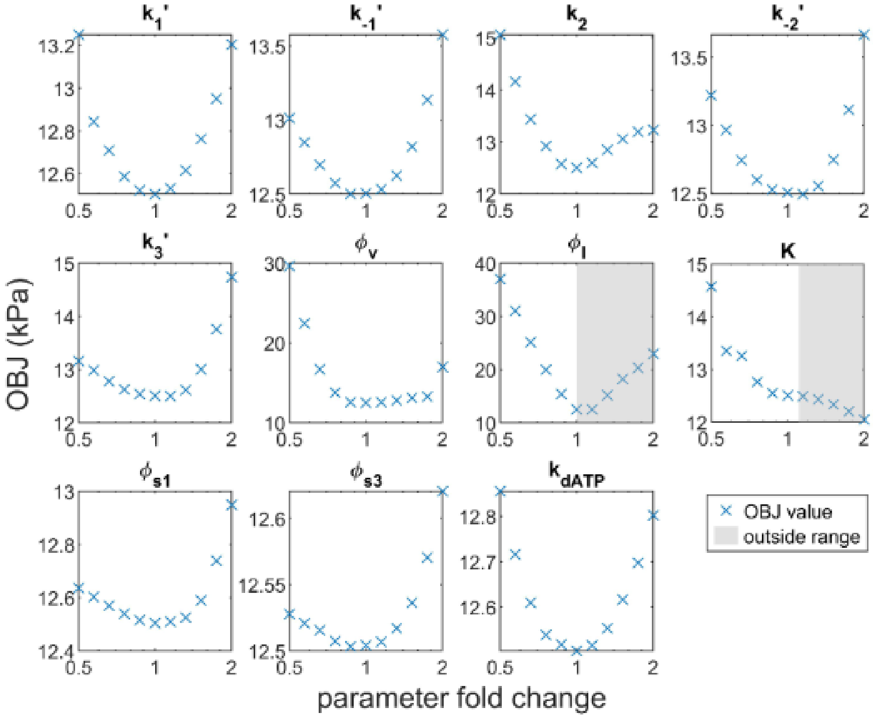

Figure A1: Profile likelihood analysis of the 11 fitted parameters of the cross-bridge model. Each subplot shows the optimal objective values (Eq. 15) obtained when the given parameter was shifted from its optimal value. Note the log-scale of the x-axes. Blue crosses show the objective values and grey shaded regions indicate parameter values that were outside the range allowed in the original optimisation.

- The authors do not explicitly discuss boundary conditions or constraints to ensure physiological behavior of the model, particularly for the strain-dependent exponential terms in Equations 9 and 12.

Following on from other recognised cross-bridge models in the field (Razumova *et al.*, 1999; Rice *et al.*, 2008; Land *et al.*, 2017; etc) we have continued using exponential terms for strain dependence. These strain-dependent terms are implemented to ensure that cross-bridge strain does not become unphysiologically high and, therefore, boundary conditions are not required for cross-bridge strain. While this relationship seems to suggest very high detachment rates, it preserves physiological behaviour of the model, by increasing the detachment rate when cross-bridge strain becomes very high. The continuous nature of these functions means that extreme behaviour is not met under typical simulation conditions, as demonstrated below.

Under work-loop simulations, strain in both states varied up to a maximum of 2.2 nm from the equilibrium (20 % of the magnitude of power stroke strain). For the strain-dependent rate with the highest sensitivity to strain (k_1 in the non-diabetic model), this corresponds to slightly less than a two-fold change in the detachment rate.

- The model does not include the more recently characterized myosin structural states like SRX and DRX that represent different energy-consuming conformations of detached myosin heads. This is a simplification that may affect the model's ability to fully capture energetic aspects of myosin function, particularly since the SRX state is associated with reduced ATP consumption and has been implicated in several cardiomyopathies. Given the paper's focus on mechanoenergetics, the potential relevance of SRX/DRX dynamics to diabetes-related cardiac dysfunction deserves a discussion.

We thank the referee for raising this interesting discussion point. Inclusion of an SRX state was not warranted, given the data available. As discussed above, our 11-parameter cross-bridge model is identifiable and well-constrained by our dataset. However, we do agree that SRX could play an interesting role in mechanoenergetics simulations and have added a short discussion point around this topic (lines 639-646).

"In recent years, there has been increased interest in the super-relaxed (SRX) state of myosin, which exhibits very low rates of ATP consumption. The behaviour of this state and its influence on cross-bridge mechanics cannot currently be well-characterised in routine mechanical experiments and has yet to be measured in human diabetic cardiac tissue. For these reasons, and to avoid unnecessary complexity, the SRX was not included in this modelling study, but its potential impact on mechanoenergetics may be important. While some cross-bridge models incorporating the effects of this state have been proposed (Campbell et al., 2018; Margara et al., 2022), there is still much to be learnt about the characterisation of SRX myosin and its effects on mechanoenergetics. Given the results of this study, future work to characterise its effect in diabetic muscle may reveal some interesting insights."

- The authors reject rapid equilibrium binding for P_1 citing "poorer fits" without quantifying this difference or providing mechanistic rationale for why direct incorporation at k_{-1} is more appropriate than at k_{-2}.

As discussed in our methods, before selecting the final form of the model, we completed the parameter fitting protocol for 64 permutations of the cross-bridge model, following methods

described in Musgrave *et al.* (2024). For the referee's interest, we include here the full results from this process, representing the normalised RMSE (in %) with columns representing ATP and P_i binding (M1 = direct incorporation, M2 = rapid equilibrium) and rows representing strain dependencies.

	1<br>(A) Pi:M1,ATP:M1	2<br>(B) Pi:M2,ATP:M1	3<br>(C) Pi:M1,ATP:M2	4<br>(D) Pi:M2,ATP:M2
1 None	4.6400	6.1900	4.4300	5.7100
2 k_1	4.3200	6.0800	3.5200	5.5100
3 k_{-1}	4.4300	6.1900	3.9900	5.6100
4 k_2	4.6000	5.9700	4.6000	5.8900
5 k_{-2}	4.6200	5.9500	4.6900	5.7100
6 k_3	4.2600	5.9200	3.6500	4.6400
7 k_1 and k_{-1}	4.3200	6.0800	3.5200	5.2500
8 k_1 and k_2	4.3100	5.9500	3.5200	5.5100
9 k_1 and k_{-2}	4.3200	5.9500	3.5200	5.5100
10 k_1 and k_3	4.2500	5.9200	3.3200	4.6400
11 k_{-1} and k_2	4.4200	5.9700	4.6000	5.6100
12 k_{-1} and k_{-2}	4.4300	5.9500	4.6600	5.6100
13 k_{-1} and k_3	4.2600	5.9100	3.5500	4.6400
14 k_2 and k_{-2}	4.8500	5.9400	4.4400	5.7100
15 k_2 and k_3	4.2700	6.3900	3.7400	4.6300
16 k_{-2} and k_3	4.2600	5.7900	3.6600	4.6400

The highlighted square shows our final model, which had the best NRMSE (3.32%) and directly to the right, the version with rapid equilibrium for P_i which is substantially higher (4.64%).

In our previous study, incorporation of P_i at the k_{-1} location was identified as the most suitable direct binding option; this was based on previous modelling studies (in particular Offer & Ranatunga (2020), who found that P_i release before tension generation was most appropriate). Given that there are also experimental studies suggesting P_i release should come later in the cycle, we additionally confirmed that this did not provide a better fit for the human data used in this model (NRMSE of more than 4 %).

We have expanded our discussion of these aspects to be more specific about why we settled on our final model (lines 626-632).

"Although rapid equilibrium binding for Pi has been supported by evidence (Musgrave et al., 2024a; Houdusse and Sweeney, 2016; Offer and Ranatunga, 2020), its more complex effects led to poorer fits for both non-diabetic and diabetic models (approximately 50 % higher objective function values, despite adding an extra parameter) and it was consequently excluded from the final human model. Additionally, to align with evidence suggesting that Pi can remain attached beyond the power stroke (Woody et al., 2019; Moretto et al., 2022), we explored incorporating Pi directly at the reverse power stroke step k−2, rather than the unbinding step k−1. However, this approach produced a worse fit to the diabetic data (over 25 % higher objective function values) and was hence rejected."

References (not including those from quotes of the manuscript)

Chung CS, Hoopes CW & Campbell KS (2017). Myocardial relaxation is accelerated by fast stretch, not reduced afterload. *J Mol Cell Cardiol* **103,** 65–73.

Han J-C, Tran K, Nielsen PMF, Taberner AJ & Loiselle DS (2014). Streptozotocin-induced diabetes prolongs twitch duration without affecting the energetics of isolated ventricular trabeculae. *Cardiovasc Diabetol* **13,** 1–16.

Jones TLM, Kaur S, Kang N, Ruygrok PN & Ward M-L (2023). Impaired calcium handling mechanisms in atrial trabeculae of diabetic patients. *Physiol Rep*; DOI: 10.14814/phy2.15599.

Kong W & Fast VG (2014). The role of dye affinity in optical measurements of Cai2+ transients in cardiac muscle. *Am J Physiol - Hear Circ Physiol* **307,** H73.

Land S, Park-Holohan SJ, Smith NP, dos Remedios CG, Kentish JC & Niederer SA (2017). A model of cardiac contraction based on novel measurements of tension development in human cardiomyocytes. *J Mol Cell Cardiol* **106,** 68–83.

Musgrave JH, Han J-C, Ward M-L, Taberner AJ & Tran K (2024). Analysis of metabolite and strain effects on cardiac cross-bridge dynamics using model linearisation techniques. *Front Physiol*; DOI: 10.3389/fphys.2023.1323605.

Offer G & Ranatunga KW (2020). The Location and Rate of the Phosphate Release Step in the Muscle Cross-Bridge Cycle. *Biophys J*; DOI: 10.1016/j.bpj.2020.09.004.

Razumova M V., Bukatina AE & Campbell KB (1999). Stiffness-distortion sarcomere model for muscle simulation. *J Appl Physiol* **87,** 1861–1876.

Rice JJ, Wang F, Bers DM & De Tombe PP (2008). Approximate model of cooperative activation and crossbridge cycling in cardiac muscle using ordinary differential equations. *Biophys J* **95,** 2368–2390.

Dear Dr Musgrave,

Re: JP-RP-2025-288463R1 "Metabolite-sensitive cross-bridge models of human atria reveal the impact of diabetes on muscle mechanoenergetics" by Julia H Musgrave, June-Chiew Han, Marie-Louise Ward, Andrew Taberner, and Kenneth Tran

We are pleased to tell you that your paper has been accepted for publication in The Journal of Physiology.

The last Word (or similar) version of the manuscript provided will be used by the Production Editor to prepare your proof. When this is ready you will receive an email containing a link to Wiley's Online Proofing System. The proof should be thoroughly checked and corrected as promptly as possible.

Authors should note that it is too late at this point to offer corrections prior to proofreading. Major corrections at proof stage, such as changes to figures, will be referred to the Editors for approval before they can be incorporated. Only minor changes, such as to style and consistency, should be made at proof stage. Changes that need to be made after proof stage will usually require a formal correction notice.

All queries at proof stage should be sent to: TJP@wiley.com.

Following copyediting and typesetting the accepted version will be published online.

Yours sincerely,

Bjorn Knollmann
Senior Editor
The Journal of Physiology

IMPORTANT POINTS TO NOTE FOLLOWING YOUR PAPER'S ACCEPTANCE:

If you would like to receive our 'Research Roundup', a monthly newsletter highlighting the cutting-edge research published in The Physiological Society's family of journals (The Journal of Physiology, Experimental Physiology, Physiological Reports, The Journal of Nutritional Physiology and The Journal of Precision Medicine: Health and Disease), please click this link, fill in your name and email address and select 'Research Roundup':
https://www.physoc.org/journals-and-media/membernews

• TRANSPARENT PEER REVIEW POLICY: To improve the transparency of its peer review process, The Journal of Physiology publishes online as supporting information the peer review history of all articles accepted for publication. Readers will have access to decision letters, including Editors' comments and referee reports, for each version of the manuscript as well as any author responses to peer review comments. Referees can decide whether or not they wish to be named on the peer review history document.

• You can help your research get the attention it deserves! Check out Wiley's free Promotion Guide for best-practice recommendations for promoting your work at: www.wileyauthors.com/eeo/guide. You can learn more about Wiley Editing Services which offers professional video, design, and writing services to create shareable video abstracts, infographics, conference posters, lay summaries, and research news stories for your research at: www.wileyauthors.com/eeo/promotion.

• IMPORTANT NOTICE ABOUT OPEN ACCESS: To assist authors whose funding agencies mandate public access to published research findings sooner than 12 months after publication, The Journal of Physiology allows authors to pay an Open Access (OA) fee to have their papers made freely available immediately on publication.

The Corresponding Author will receive an email from Wiley with details on how to register or log-in to Wiley Authors Services where you will be able to place an order.

• You can check if your funder or institution has a Wiley Open Access Account here: https://authorservices.wiley.com/author-resources/Journal-Authors/licensing-and-open-access/open-access/author-compliance-tool.html.

EDITOR COMMENTS

Reviewing Editor:

Your manuscript was assessed again and all expert referees confirmed that their concerns have been addressed sufficiently.

Senior Editor:

The MS is now acceptable. Thank you for an excellent contribution to the Journal!

REFEREE COMMENTS

Referee #1:

The authors responded appropriately to the prior comments. No further issues.

Referee #2:

The authors have addressed all previously raised concerns, demonstrating careful attention to the reviewer feedback. I commend the authors for their relevant work and diligent revisions.
